# Agreement among Health Care Professionals in Diagnosing Case Vignette-Based Surgical Site Infections

**DOI:** 10.1371/journal.pone.0035131

**Published:** 2012-04-17

**Authors:** Didier Lepelletier, Philippe Ravaud, Gabriel Baron, Jean-Christophe Lucet

**Affiliations:** 1 Infection Control Unit, Bichat-Claude Bernard Hospital, Assistance Publique-Hôpitaux de Paris, Paris, France; 2 University Paris Diderot, Sorbonne Paris Cité, Paris, France; 3 Hôtel Dieu, Centre d'Épidémiologie Clinique, Assistance Publique des Hôpitaux de Paris, Paris, France; 4 INSERM, U738, Paris, France; 5 University Paris Descartes, Sorbonne Paris Cité, Paris, France; University of Maryland, United States of America

## Abstract

**Objective:**

To assess agreement in diagnosing surgical site infection (SSI) among healthcare professionals involved in SSI surveillance.

**Methods:**

Case-vignette study done in 2009 in 140 healthcare professionals from seven specialties (20 in each specialty, Anesthesiologists, Surgeons, Public health specialists, Infection control physicians, Infection control nurses, Infectious diseases specialists, Microbiologists) in 29 University and 36 non-University hospitals in France. We developed 40 case-vignettes based on cardiac and gastrointestinal surgery patients with suspected SSI. Each participant scored six randomly assigned case-vignettes before and after reading the SSI definition on an online secure relational database. The intraclass correlation coefficient (ICC) was used to assess agreement regarding SSI diagnosis on a seven-point Likert scale and the kappa coefficient to assess agreement for superficial or deep SSI on a three-point scale.

**Results:**

Based on a consensus, SSI was present in 21 of 40 vignettes (52.5%). Intraspecialty agreement for SSI diagnosis ranged across specialties from 0.15 (95% confidence interval, 0.00–0.59) (anesthesiologists and infection control nurses) to 0.73 (0.32–0.90) (infectious diseases specialists). Reading the SSI definition improved agreement in the specialties with poor initial agreement. Intraspecialty agreement for superficial or deep SSI ranged from 0.10 (−0.19–0.38) to 0.54 (0.25–0.83) (surgeons) and increased after reading the SSI definition only among the infection control nurses from 0.10 (−0.19–0.38) to 0.41 (−0.09–0.72). Interspecialty agreement for SSI diagnosis was 0.36 (0.22–0.54) and increased to 0.47 (0.31–0.64) after reading the SSI definition.

**Conclusion:**

Among healthcare professionals evaluating case-vignettes for possible surgical site infection, there was large disagreement in diagnosis that varied both between and within specialties.

## Introduction

Surgical site infection (SSI) is receiving considerable interest from healthcare authorities, the media, and the public. Because they are often considered avoidable, the SSI rate has been used for performance assessments and benchmarking [1], and several countries require that healthcare facilities publish SSI rates to improve transparency, and possibly quality of care and patient safety [2]. However, the evidence that publishing quality indicators improves care is scant [3]. Recent reports indicate a need for improved measurement reliability [4], and mandatory public reporting remains a focus of vigorous debate [5], [6].

Methodological issues, related to benchmarking and public reporting, remain controversial. If the SSI rate is to serve as a performance indicator, then valid and consistent SSI rates must be obtained [2]. SSI rates vary according to co-morbidities, to the contamination class and conditions of the surgical procedure. The need for adjustment has been demonstrated, and most surveillance networks use risk stratification [7], [8]. Another factor that influences SSI rates is the certainty of SSI diagnosis. The extent to which different healthcare professionals will agree regarding the diagnosis of SSI depends on many factors including training, experience, and the use of a common SSI definition. A single-centre study showed variability in the SSI incidence rate according to the SSI definition [9].

We designed a study to assess agreement among healthcare professionals within and among different specialties regarding diagnosis and superficial or deep SSI, based on case-vignettes concerning real patients. We also evaluated whether the providing of NHSN criteria change the agreement estimates

## Methods

### Development of the case-vignettes

Case-vignettes allow an assessment of the same cases by healthcare professionals involved in diagnosing and treating SSI. We used blinded random assignment of the case-vignettes to healthcare professionals.

We followed consecutive patients with suspected SSI throughout their hospitalization or re-hospitalization in four surgical units, two digestive surgery units and two cardiac surgery units in three French University hospitals. Each day, a bedside evaluation was performed; the medical chart and nurses' log were reviewed; and the findings from laboratory and microbiology tests, and imaging studies were recorded. Photographs of the wound and/or computed tomography (CT) results were obtained. We identified 40 patients with suspected SSI and complete information, 20 in cardiac surgery and 20 with gastrointestinal surgery (colorectal or bariatric procedures).

Suspected SSI was defined as wound modification or discharge and/or evidence of infection. We used the Centers for Disease Control SSI definition (Table S1) [10], which is identical to the European HELICS/IPSE definition [11], [12].

### Participants

We identified 20 healthcare professionals from each of seven specialties potentially involved in SSI management: surgeons in any specialty, anaesthesiologists, microbiologists, infectious diseases specialists, infection control nurses, infection control physicians, and public health specialists.

To build our study sample, participants were recruited by direct solicitation of close colleagues from other hospitals and relation network. In addition, we used the French network for SSI surveillance for surgeons' identification, together with several French societies for the other specialties, i.e. the Public Health Society, the French Hygiene Society, the French Society for Infectious Diseases, the French Society for Microbiology and the French Society for Anesthesiology and Intensive Care.

No randomized selection was done and the first 20 participants volunteering to participate were included in the study. Most of the participants were health-care workers as some of public health specialists were engineer involved in the risk control in hospitals. All 140 participants worked full time in public or private French hospitals with surgical activity, including university and non-university facilities. None of them had been involved in the management of patients used to build the vignettes. All the 140 participants scored the assigned case vignettes during a 4-month period. Because of the observational and blinded nature of the study, the institutional review board of the Bichat-Claude Bernard Hospital waived the requirement for informed consent.

### Study design and data

Twenty of the 40 vignettes were randomly assigned for assessing the intra-specialty agreement. These twenty vignettes were scored twice without the SSI definition by participants for each specialty. The same 20 vignettes were also scored twice with the SSI definition by participants inside each specialty. All 40 case vignettes were randomly assigned for assessing the inter-specialty reliability of scoring with or without the SSI definition. In total, each participant scored six vignettes. The first three vignettes were scored without the SSI definition. Then three other vignettes were scored with the SSI definition. Of the 6 vignettes read by one participant, 5 were different, and one was scored twice, first without the SSI definition then with the SSI definition. In total, 20 vignettes were read four times and 20 vignettes were read two times by specialty. Consequently, taking into account the seven specialties, 20 vignettes were scored 28 times and 20 vignettes were scored 14 times, for a theoretical total of 840 scores.

Scores were assigned using a seven-point Likert scale ranging from “SSI certainly absent” (score one) to “SSI certainly present” (score seven) [13]. When the score was between four and seven, the participant scored superficial/deep SSI on a three-point scale (one, superficial SSI; two, depth unclear; and three, deep or organ/space-related SSI). We simplified the depth assessment by putting deep and organ/space-related SSIs in the same group, as both SSI categories have the same severe consequences in terms of mortality, morbidity, and prolongation of hospital stay.

An online secure relational database was constructed for collecting the study data. Each participant had a personal login and password [14], [15]. The patient data were presented chronologically, and the scores assigned before reading the SSI definition could not be changed. Before scoring the vignettes, each participant provided the following information: age, gender, type of hospital, and duration of experience in the current job.

### Statistical analysis

We estimated the number of vignettes and participants needed to assess agreement within specialties, according to the precision of the intraclass correlation coefficient [16] and taking into account the feasibility of the study. If 20 vignettes were scored twice and if the expected coefficient is close to 0.60, then the semi-width of the exact 95 per cent confidence interval (i.e., the precision) is equal to 0.29.

Data were described as mean ± SD, median (interquartile range), or percentage.

Intra- and interspecialty agreement analysis were performed before and after reading the SSI definition. To evaluate intra- and interspecialty agreement regarding the one-seven Likert scale, we computed the intraclass correlation coefficient (ICC). We used the bootstrap procedure (Bias-corrected and accelerated bootstrap) to estimate 95% confidence intervals (95%CIs). An ICC value of 0 indicates the level of agreement produced by chance alone and a value of 1 indicates perfect agreement. We defined poor agreement as ICC values lower than 0.4, good agreement as ICC values of 0.4 to 0.7, and very good agreement as ICC values higher than 0.7 [17].

We also dichotomized the Likert scale (i.e. scores one to four, corresponding to the absence of SSI and scores five to seven, corresponding to the presence of SSI). To evaluate intraspecialty agreement, observed agreement (exact 95% confidence intervals) and simple kappa coefficient (with 95% confidence intervals) were computed. To evaluate interspecialty agreement, we computed kappa for multiple raters with their 95%CIs [18]. Agreement assessed by Kappa coefficient is considered poor when kappa is 0.20 or less, fair when kappa is 0.21–0.40, moderate when kappa is 0.41–0.60, good when kappa is 0.61–0.80 and very good when the kappa value is 0.81–1.00 [19].

To evaluate intra- and interspecialty agreement regarding superficial/deep SSI scored on the 3-point scale, we computed observed agreement (exact 95% confidence intervals) and kappa coefficient (with 95% confidence intervals). We added a fourth category comprising the participants who did not score SSI depth because their score for SSI diagnosis on the 7-point Likert scale was lower than 4.

Analyses was performed using SAS System, Version 9.2 (SAS Institute, Cary, NC) for descriptive and kappa statistics and graphs. R 1.9 software and its “boot” and “psy” library were used for computing ICCs.

## Results

### Characteristics of the participants and case-vignettes

Table S2 reports the main characteristics of the 140 participants. All 140 participants completed the study. They originated from 29 University and 36 non University hospitals in France. There was one participant in 40 hospitals (62%), 2 to 4 participants in 20 (31%) hospitals and 5 or more participants in 5 (7%) hospitals. Their median (IQR) age was 48 (29–65) years and 77 (55%) were male. Their median time in their current job was 17 (1–36) years and 98 (70%) of them were directly involved in SSI surveillance programs in their healthcare facility. Among the 140 participants, 104 (74%) worked in publicly funded healthcare facilities, 19 (14%) in private healthcare facilities, and 17 (12%) in other types of centers.

SSI was suspected before hospital discharge in 36 patients and after hospital discharge in 4 patients, who required re-admission. Wound modification was a feature in all 20 cardiac surgery patients and in 11 (55%) gastrointestinal surgery patients. Microbiological specimens were obtained from the surgical wound in all 20 cardiac surgery patients and were positive in 11 (55%) of these patients. Of the 20 gastrointestinal surgery patients, 3 underwent wound sampling for microbiological tests, which were positive in 2 patients. Based on the consensus of the two main investigators (DLP and JCL), there was an agreement in 36 out of the 40 vignettes, with the presence of SSI in 21 vignettes (52.5%).

### Case-vignette scores

In total, the 40 case-vignettes were scored 822 times and not 840 as theoretically scheduled. Due to a computer assignment glitch, three surgeons were assigned vignettes that had previously been assigned to other surgeons. Therefore, the 18 vignettes that these surgeons were supposed to receive were not scored. The median SSI diagnosis score before reading the SSI definition on the seven-point Likert scale varied across specialties from four (IQR, 2–6) for public health specialists and infection control nurses to seven for anesthesiologists (IQR, 3.5–7) (Table 1).

**Table 1:** Distribution of scores assigned before reading the definition of surgical site infection, on a 7-point Likert scale, in each of the seven specialties.

Specialty	Number of vignettes scored*	Mean score (SD)	Median score (IQR)	Min. - Max.
Anesthesiologist	40	5.4 (2.2)	7.0 (3.5–7.0)	1.0–7.0
Surgeon	34**	4.8 (2.3)	5.5 (2.0–7.0)	1.0–7.0
Public health specialist	40	4.1 (2.0)	4.0 (2.0–6.0)	1.0–7.0
Infection control physician	40	4.8 (2.3)	6.0 (2.0–7.0)	1.0–7.0
Infection control nurse	40	4.1 (2.2)	4.0 (2.0–6.0)	1.0–7.0
Infectious diseases specialist	40	4.9 (2.3)	6.0 (2.0–7.0)	1.0–7.0
Microbiologist	40	4.1 (2.4)	4.5 (2.0–6.0)	1.0–7.0

SD, standard deviation; IQR, interquartile range; min, minimum; max, maximum.

* Number of vignettes scored (20 vignettes were scored twice for each specialty).

** missing values due a computer assignment glitch.

### Intraspecialty and interspecialty agreement regarding SSI diagnosis

The intraspecialty ICC based on scores assigned without the SSI definition ranged across specialties from 0.15 to 0.73. Agreement was very good among infectious diseases specialists (ICC, 0.73; 95%CI, 0.32–0.90); good among surgeons (0.45, 0.00–0.81), public health specialists (0.56, 0.18–0.80) and microbiologists (0.56, 0.19–0.81); and poor among infection control physicians (0.30, 0.00–0.69), anesthesiologists (<0.20, 0.00–0.59), and infection control nurses (<0.20) (Table 2). Scoring with the SSI definition improved agreement only within the specialties where agreement was poor initially (Table 2).

**Table 2:** Assessment of surgical site infection (SSI) diagnosis for 40 vignettes (20 cardiac surgery cases and 20 gastrointestinal surgery cases) developed based on real patients in three French university hospitals.

	SSI diagnosis score, 7-point Likert scale (Intraclass correlation coefficient)			
	Number of vignettes scored*	Scoring without the SSI definition (95%CI)	Number of vignettes scored*	Scoring with the SSI definition (95%CI)
**Intraspecialty correlation**				
Anesthesiologist	40	0.15 (0.00–0.59)	40	0.35 (0.00–0.73)
Surgeon	32**	0.45 (0.00–0.81)	28**	0.42 (0.09–0.80)
Public health specialist	40	0.56 (0.18–0.80)	40	0.29 (0.00–0.66)
Infection control physician	40	0.30 (0.00–0.69)	40	0.01 (0.00–0.48)
Infection control nurse	40	0.19 (0.00–0.59)	40	0.56 (0.00–0.80)
Infectious diseases specialist	40	0.73 (0.32–0.90)	40	0.66 (0.22–0.91)
Microbiologist	40	0.56 (0.19–0.81)	40	0.42 (0.00–0.71)
**Interspecialty correlation**	238**	0.36 (0.22–0.54)	238**	0.47 (0.31–0.64)

* Number of vignettes scored (for intraspecialty 20 vignettes were scored twice and for interspecialty 34 vignettes were scored 7 times).

** missing values due a computer assignment glitch.

After dichotomization, results were similar with good agreement among infectious diseases specialists (0.66, 0.30–1.00), moderate agreement among microbiologists (0.60, 0.26–0.94) and public health specialists (0.52, 0.20–0.84), fair agreement among surgeons (0.38, −0.05–0.80) and infection control physicians (0.21, −0.24–0.64) and poor agreement in other specialties (Table 3).

**Table 3:** Assessment of surgical site infection (SSI) diagnosis for 40 vignettes (20 cardiac surgery cases and 20 gastrointestinal surgery cases) developed based on real patients in three French university hospitals.

	SSI diagnosis score, 7-point Likert scale categorized in 2 classes (1,2,3,4 vs 5,6,7)		
	Scoring without the SSI definition (95%CI)		
	Number of vignettes scored*	Observed agreement (%) (95%CI)	Kappa coefficient (95%CI)
**Intraspecialty**			
Anesthesiologist	40	65.0 (40.8–84.6)	0.15 (−0.28–0.57)
Surgeon	32**	68.8 (41.3–89.0)	0.38 (−0.05–0.80)
Public health specialist	40	75.0 (50.9–91.3)	0.52 (0.20–0.84)
Infection control physician	40	65.0 (40.8–84.6)	0.21 (−0.24–0.64)
Infection control nurse	40	55.0 (31.5–76.9)	0.12 (−0.30–0.53)
Infectious diseases specialist	40	85.0 (62.1–96.8)	0.66 (0.30–1.00)
Microbiologist	40	80.0 (56.3–94.3)	0.60 (0.26–0.94)
**Interspecialty**	238**	-	0.28 (0.21–0.36)
	**Scoring with the SSI definition (95%CI)**		
**Intraspecialty**			
Anesthesiologist	40	75.0 (50.9–91.3)	0.43 (0.01–0.85)
Surgeon	28**	71.4 (41.9–91.6)	0.28 (−0.05–0.63)
Public health specialist	40	65.0 (40.8–84.6)	0.30 (−0.06–0.66)
Infection control physician	40	55.0 (31.5–76.9)	−0.03 (−0.45;0.41)
Infection control nurse	40	65.0 (40.8–84.6)	0.40 (−0.01–0.80)
Infectious diseases specialist	40	85.0 (62.1–96.8)	0.62 (0.25–1.00)
Microbiologist	40	70.0 (45.7–88.1)	0.41 (0.02–0.80)
**Interspecialty**	238**	-	0.41 (0.34–0.48)

* Number of vignettes scored (for intraspecialty 20 vignettes were scored twice and for interspecialty 34 vignettes were scored 7 times).

** missing values due a computer assignment glitch.

Scoring without the SSI definition, the interspecialty ICC was 0.36 (0.22–0.54). Scoring with the definition improved the ICC to 0.47 (0.31–0.64) (Table 2).

### Agreement regarding SSI depth

Intraspecialty kappa values for superficial/deep SSI scored without the SSI definition varied from 0.10 to 0.54 (Table 4). Agreement was moderate among surgeons (k, 0.54, 0.25–0.83); fair among public health specialists (0.32, 0.06–0.59), infection control physicians (0.25, −0.04–0.55), infectious diseases specialists (0.22, 0.04–0.47), and microbiologists (0.21, −0.05–0.46); and poor within other specialties. Reading the SSI definition was followed by an increase in the intraspecialty kappa values mainly among the infection control nurses (Table 4).

**Table 4:** Assessment of surgical site infection (SSI) depth for 40 vignettes (20 cardiac surgery cases and 20 gastrointestinal surgery cases) developed based on real patients in three French university hospitals.

	Number of vignettes scored*	Depth SSI not scored (n, %)***	Kappa coefficient (95%CI)
	**Scoring without the SSI definition**		
**Intraspecialty correlation**			
Anesthesiologist	40	9 (22.5)	0.13 (−0.14–0.39)
Surgeon	32**	11 (34.4)	0.54 (0.25–0.83)
Public health specialist	40	15 (37.5)	0.32 (0.06–0.59)
Infection control physician	40	13 (32.5)	0.25 (−0.04–0.55)
Infection control nurse	40	20 (50.0)	0.10 (−0.19–0.38)
Infectious diseases specialist	40	10 (25.0)	0.22 (0.04–0.47)
Microbiologist	40	18 (45.0)	0.21 (−0.05–0.46)
**Interspecialty correlation**	238**	88 (37.0)	0.21 (0.16–0.25)
	**Scoring with the SSI definition**		
**Intraspecialty correlation**			
Anesthesiologist	40	13 (32.5)	0.24 (−0.03–0.51)
Surgeon	28**	12 (30.0)	0.38 (0.07–0.70)
Public health specialist	40	12 (30.0)	0.15 (−0.13–0.44)
Infection control physician	40	13 (32.5)	0.11 (−0.15–0.38)
Infection control nurse	40	16 (40.0)	0.41 (−0.09–0.72)
Infectious diseases specialist	40	14 (35.0)	0.20 (−0.07–0.48)
Microbiologist	40	17 (42.5)	0.10 (−0.14–0.33)
**Interspecialty correlation**	238**	92 (38.7)	0.29 (0.27–0.31)

SSI Depth was scores on a 4-point scale: scale 1, no SSI in vignettes with scores lower than 4 on the 7-point Likert for the absence/presence of SSI; scale 2, superficial SSI; scale 3, uncertainty about SSI diagnosis, scale 4, deep/organ space SSI in vignettes scored 4 or more on the 7-point Likert scale.

* For intraspecialty 20 vignettes were scored twice and for interspecialty 34 vignettes were scored 7 times).

** missing values due a computer assignment glitch.

*** Depth was not scored because scores of the absence/presence of SSI were lower than 4 on the 7-point Likert scale.

Interspecialty kappa values for SSI depth scored before reading the SSI definition were 0.21 (0.16–0.25). Reading the SSI definition increased in the interspecialty kappa values to 0.29 (0.27–0.31).

## Discussion

In a large panel of healthcare professionals from different specialties involved in SSI surveillance, agreement regarding the diagnosis and depth assessment of SSI varied across specialties and across individuals within each specialty. Scoring with the SSI definition improved agreement regarding the SSI diagnosis and depth assessment only in the specialties where agreement was poor initially.

There is an abundance of studies evaluating SSI risk factors and risk stratification [20]. In addition, many studies assessed techniques designed to improve the measurement of the numerator, i.e., the number of SSIs. The reference standard method for SSI surveillance includes daily bedside surveillance and post-discharge surveillance [21]. Several authors evaluated the usefulness of surrogate indicators [22], [23].

We are aware of a single study evaluating the impact of different SSI definitions on SSI rates [9]. In this study, SSI rates varied by more than 50% when small changes were made in the SSI definition. This study has limitations, however, including the single-centre design and possible observation bias due to the expectation that SSI rates would vary according to the SSI definition. Other studies suggest imperfect agreement across physicians regarding the diagnosis of SSI. In one study, wide differences in the diagnosis of SSI were noted between infection control practitioners and surgeons, as well as across surgeons [24]. A recent study showed that surgeons tended to diagnose only deep and organ space SSIs, whereas the infection control team doubled the number of SSIs by also detecting superficial SSIs [25]. A study comparing SSI rates from 11 European countries showed substantial differences in SSI distribution, with the proportion of superficial SSIs ranging from 80% to 20%–30%, suggesting differences in SSI detection and/or classification across countries [26].

Our study further supports the existence of considerable uncertainty regarding the detection of SSI. Providing the SSI definition did not change the agreement, except in specialties with an initially low agreement. Agreement decreased in infection control physician, without clear explanation. Our results are probably reliable, as we placed the participants in unbiased conditions by asking them to score the same case-vignettes through an Internet database. This method ensured that the participants were not influenced by factors such as perceived SSI risk in a particular unit or patient. Considering such factors would likely have increased disagreement among participants. Thus, SSI rates may be less than ideal performance indicators. In addition, mandatory surveillance and public reporting may lead to gaming, misinterpretation, and underreporting [5], [6]. As recently suggested, there is a need for regular assessments of the reliability and validity of infection reporting [27].

We found scoring differences across participants and across types of case-vignettes. As expected, agreement for diagnosis and superficial/deep SSI assessment were well correlated among surgeons. More surprisingly, the correlation was poor among infection control professionals. Our results further support the need for a multidisciplinary approach to SSI surveillance [28].

Our study has several limitations. First, only one investigator (DLP) selected the suspected SSI and standardized the vignette. In addition, each participant worked alone to determine whether SSI was present in each vignette. SSI is often a difficult diagnosis that requires discussion among surgeons and infection control professionals. The main goal of SSI surveillance is accurate SSI rate determination with feedback of appropriate data to surgeons, but another goal is to strengthen collaboration between surgical and infection-control teams in order to implement effective preventive strategies and to improve quality of care. Our results indicate that surveillance should not be performed by individuals in a single specialty [28]. Second, the participants scored vignettes via an online database. The vignettes were built from real cases, and the diagnosis of SSI may have been easier for healthcare professionals who had had direct contact with the patient. Third, the study was not designed to assess the accuracy of SSI diagnosis. Instead, we focused on agreement among healthcare professionals regarding SSI diagnosis. The two main investigators tentatively classified the vignettes as indicating SSI or no SSI, but their classification differed for several vignettes. We were therefore unable to determine which participants made the right diagnosis. This is illustrated in Table 1, which shows SSI diagnosis score differences of up to 6 points between two participants from the same specialty. Fourth, we selected suspicions of SSI to assess the agreement in the diagnosis of SSI. SSI suspicion however occurs in a small proportion of patients after surgical procedure. The agreement about the presence of SSI would have been higher if the heterogeneity of the population had been greater, e.g., if the population studied have been an actual series of surgical patients rather than a series of surgical patients with suspected infection. Fifth, the study was performed in a country where specific SSI surveillance method and practice are used. Results might have been different in another country. Finally, we selected case-vignettes in only two surgical specialties, representing clean and contaminated surgery, respectively. Increasing the spectrum of surgical procedures would probably have increased the degree of disagreement regarding SSI diagnosis and depth assessment. For example, SSI may be particularly difficult to diagnose in the absence of a skin incision, e.g., after vaginal hysterectomy or transurethral resection of the prostate.

In conclusion, among healthcare professionals evaluating case-vignettes for possible surgical site infection, there was large disagreement in diagnosis that varied both between and within specialties. These results support a multidisciplinary approach for SSI diagnosis. Our finding supports the need for caution when using SSI rates for benchmarking or requiring public reporting of SSI rates. Similar concerns have been voiced regarding other publicly reported infection rates, such as rates of catheter-related bloodstream infections [29], [30] or ventilator-associated pneumonia [31] in critically ill patients. Nevertheless, SSI surveillance and feedback remain important tools for SSI prevention [32]. Further studies are needed to improve agreement regarding the diagnosis of SSI.

## Supporting Information

Table S1 **This table presents the definition of surgical site infection from the Centers of Diseases Control and Prevention (CDC) that were used in this study** [**10**] **.**
(DOC)

Table S2 * This time was calculated from the date of medical graduation (MD and PharmD) and was not calculated for nurses or other professionals.
(DOC)
