# Supplementary material for: Agreement among Health Care Professionals in Diagnosing Case Vignette-Based Surgical Site Infections
Source: PLoS One. 2012 Apr 17;7(4):e35131. doi: 10.1371/journal.pone.0035131 (PMC3328479; doi:10.1371/journal.pone.0035131)
Supplement: Table S1 — This table presents the definition of surgical site infection from the Centers of Diseases Control and Prevention (CDC) that were used in this study [10] . (DOC) [file pone.0035131.s001.doc]

**Table S1:** Definition of surgical site infection used in the study by the participants to score the case vignettes [10].

| **A superficial SSI** must meet the following criterion: Infection occurs within 30 days after the operative procedure and involves only skin and subcutaneous tissue of the incision and patient has at least one of the following:  **a.** Purulent draining from the superficial incision  **b.** Organisms isolated from an aseptically obtained culture of fluid or tissue from the superficial incision  **c.** At least one of the following signs or symptoms of infection: pain or tenderness, localized swelling, redness, or heat, and superficial incision is deliberately opened by surgeon, unless incision is culture-negative  **d.** Diagnosis of superficial incisional SSI by the surgeon or attending physician  *REPORTING INSTRUCTIONS:*  *• Do not report a stitch abscess (minimal inflammation and discharge confined to the points of suture penetration) as an infection*  *• Do not report a localized stab wound infection as SSI, instead report as skin or soft tissue infection, depending on its depth.* |
| --- |
| **A deep incisional SSI** must meet the following criterion:  Infection occurs within 30 days after the operative procedure if no implant is left in place or within one year if implant is in place and the infection appears to be related to the operative procedure and involves deep soft tissues (e.g., fascial and muscle layers) of the incision and patient has at least one of the following:  **a**. Purulent drainage from the deep incision but not from the organ/space component of the surgical site  **b.** A deep incision spontaneously dehisces or is deliberately opened by a surgeon when the patient has at least one of the following signs or symptoms: fever (.38° C), or localized pain or tenderness, unless incision is culture-negative  **c.** An abscess or other evidence of infection involving the deep incision is found on direct examination, during reoperation, or by histopathologic or radiologic examination  **d.** Diagnosis of a deep incisional SSI by a surgeon or attending physician  *REPORTING INSTRUCTIONS:*  *• Classify infection that involves both superficial and deep incision sites as deep incisional SSI.*  *• Report culture specimen from deep incisions as ID (incisional drainage).* |
| **An organ/space SSI** involves any part of the body, excluding the skin incision, fascia, or muscle layers, that is opened or manipulated during the operative procedure.  Infection occurs within 30 days after the operative procedure if no implant is left in place or within one year if implant is in place and the infection appears to be related to the operative procedure and infection involves any part of the body, excluding the skin incision, fascia, or muscle layers, that is opened or manipulated during the operative procedure and patient has at least one of the following:  **a.** Purulent drainage from a drain that is placed through a stab wound into the organ/space  **b.** Organisms isolated from an aseptically obtained culture or fluid or tissue in the organ/space  **c.** An abscess or other evidence of infection involving the organ/space that is found on direct examination, during reoperation, or by histopathologic or radiologic examination  **d.** Diagnosis of an organ/space SSI by a surgeon or attending physician  *REPORTING INSTRUCTIONS:*  *• Occasionally an organ/space infection drains through the incision. Such infection generally does not involve reoperation and is considered a complication of the incision. Therefore, it is classified as a deep incisional SSI.*  *• Report culture specimen from organ/space as DD (deep drainage).* |
