# Supplementary material for: Agreement among Health Care Professionals in Diagnosing Case Vignette-Based Surgical Site Infections
Source: PLoS One. 2012 Apr 17;7(4):e35131. doi: 10.1371/journal.pone.0035131 (PMC3328479; doi:10.1371/journal.pone.0035131)
Supplement: Table S2 — * This time was calculated from the date of medical graduation (MD and PharmD) and was not calculated for nurses or other professionals. (DOC) [file pone.0035131.s002.doc]

**Table S2. Characteristics of the 140 participants**

| Specialty | Anesthesiologist | Surgeon | Public Health Specialist | IC Nurse | IC Practitioner | ID Specialist | Microbiologist | Total |
| --- | --- | --- | --- | --- | --- | --- | --- | --- |
| Number of participants (%) | 20 | 20 | 20 | 20 | 20 | 20 | 20 | 140 (100) |
| Age, median (IQR) | 51 (35-64) | 48 (34-64) | 47 (32-60) | 51 (36-59) | 49 (30-62) | 43 (29-65) | 46 (29-60) | 48 (29-65) |
| Male sex, N | 17 | 15 | 8 | 1 | 12 | 15 | 9 | 77 (55) |
| Type of hospital |  |  |  |  |  |  |  |  |
| *Public hospital* | 14 | 13 | 18 | 10 | 18 | 15 | 16 | 104 (74) |
| *Private hospital* | 5 | 4 | 2 | 3 | 1 | 2 | 2 | 19 (14) |
| *Other type of centers* | 1 | 3 | 0 | 7 | 1 | 3 | 2 | 17 (12) |
| Graduation (%) |  |  |  |  |  |  |  |  |
| *Medical doctor* | 20 | 20 | 10 | - | 19 | 20 | 14 | 104 (74) |
| *Pharmacy doctor* | - | - | 0 | - | 1 | - | 6 | 7 (5) |
| *Nurse* | - | - | 6 | 20 | 0 | - | 0 | 26 (18) |
| *Other* | - | - | 4 | - | 0 | - | 0 | 4 (3) |
| Professional experience in the current job, year, median (IQR) | 22 (5-36) | 16 (2-34) | 19 (4-30)* | -* | 20 (2-34) | 13 (2-34) | 15 (1-33) | 17 (1-36) |
| Participation to SSI surveillance | 14 | 19 | 10 | 18 | 19 | 9 | 9 | 98 (70) |

* This time was calculated from the date of medical graduation (MD and PharmD) and was not calculated for nurses or other professionals.
